# Uptrend in esotropia incidence in the era of excessive smartphone use: A nationwide population-based cohort study in Japan, 2014–2019

**DOI:** 10.1371/journal.pdig.0001382

**Published:** 2026-04-22

**Authors:** Saori Wada, Manabu Miyata, Masahiro Miyake, Ai Kido, Takuro Kamei, Hiroaki Ueshima, Shinya Nakao, Akinari Yamamoto, Kenji Suda, Eri Nakano, Hiroshi Tamura, Akitaka Tsujikawa

**Affiliations:** 1 Department of Ophthalmology and Visual Sciences, Kyoto University Graduate School of Medicine, Kyoto, Japan; 2 Center for Innovative Research and Education in Data Science, Institute for Liberal Arts and Sciences, Kyoto University, Kyoto, Japan; Jordan University of Science and Technology, JORDAN

## Abstract

Acute acquired comitant esotropia (AACE) has recently garnered attention, as numerous case-series studies have reported its occurrence following excessive smartphone use. However, no large-scale epidemiological evidence of an increase in esotropia has been provided. This study aimed to investigate the change in the annual incidence of esotropia in Japan between 2014 and 2019—a period marked by a rapid increase in smartphone users. This nationwide population-based cohort study used the National Database of Health Insurance Claims and Specific Health Checkups of Japan, which covers almost the entire Japanese population. We counted the number of newly diagnosed esotropia cases and esotropia-related strabismus surgeries for each year from 2014 to 2019. Annual incidence rates were calculated by dividing these numbers by the corresponding year’s population. We also investigated the correlation between the annual incidence rate and smartphone household penetration, based on data from the Japanese Ministry of Internal Affairs and Communications. The annual incidence of esotropia gradually increased from 32.26 (95% confidence interval [CI], 31.95–32.57) to 36.61 (95% CI, 36.28–36.95) per 100,000 person-years between 2014 and 2019. The mean annual increase rate was 2.49 ± 1.62%. The number of esotropia-related strabismus surgeries also increased from 3,061 to 3,743 during the same period. The annual incidence of esotropia correlated significantly with smartphone household penetration (*P* = 0.005, r = 0.943). In conclusion, this ecological study provides the first population-based evidence of a significant uptrend in the annual incidence of esotropia and related strabismus surgeries in Japan between 2014 and 2019. The rapid increase in digital device use warrants caution and may be associated with this increase, suggesting a need for clinical guidelines on screen time.

## Author summary

Acute acquired comitant esotropia causes the eyes to suddenly cross inward, resulting in double vision. Growing clinical concern links excessive smartphone use to this condition. However, large-scale nationwide epidemiological evidence tracking the actual number of patients with esotropia remained absent. Therefore, we investigated the change in the annual incidence of esotropia in Japan between 2014 and 2019—a period marked by a rapid increase in smartphone users—using the National Database of Health Insurance Claims and Specific Health Checkups of Japan, covering nearly the entire population. We found that the annual incidence of esotropia gradually increased from 32.26 to 36.61 per 100,000 person-years between 2014 and 2019. Furthermore, the number of esotropia-related strabismus surgeries increased from 3,061 to 3,743 during the same period. The annual incidence of esotropia correlated significantly with smartphone household penetration. These results suggest that excessive near-work on digital devices exhausts the eyes’ ability to maintain proper alignment, particularly in individuals with an underlying tendency for their eyes to cross. The rapid increase in digital device use warrants caution and may be associated with this increase, suggesting a need for clinical guidelines on screen time.

## Introduction

Acute acquired comitant esotropia (AACE), a condition that causes sudden-onset diplopia, has recently garnered the attention of clinicians since a case-series study in 2016 first reported 12 patients with AACE who had a history of excessive smartphone use [1]. A multicenter questionnaire study has been performed concerning the relationship between AACE and digital device use in Japan [2,3]. Case-series studies reported that the esodeviation in patients with what we call “smartphone esotropia” was reduced by refraining from smartphone use [1,3,4]. Nevertheless, no large-scale epidemiological studies to date have shown that esotropia indeed increased in accordance with the marked increase in smartphone use in recent years.

**Fig 1:**
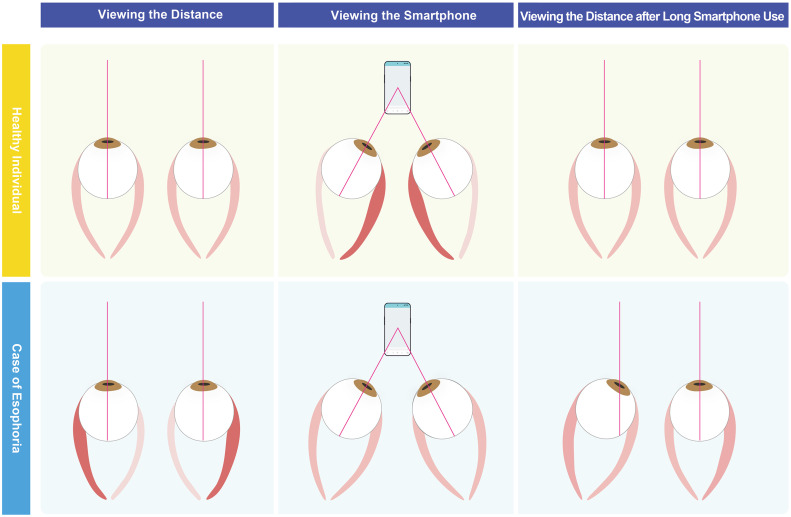
Conceptual images proposing the mechanism of esophoria to esotropia triggered by long and excessive smartphone use. This figure illustrates the hypothesized difference in ocular position and muscle tonus when viewing the distance between a healthy individual (upper row) and a case of esophoria (lower row) across three conditions: viewing the distance (left), viewing a smartphone (middle), and viewing the distance after long smartphone use (right). (Upper row) A healthy individual without ocular deviation maintains a stable power balance between the medial rectus muscle (MR) and lateral rectus muscle (LR) when viewing the distance. While long and excessive smartphone use may induce asthenopia, the balance is easily restored. (Lower row) In a case of esophoria (latent ocular deviation, absence of diplopia), LR contraction and MR relaxation must constantly work to maintain the straight gaze position (divergence) when viewing the distance. In the figure, this increased LR tonus is represented by the thickened and darker red appearance of the muscle (bottom left). This constant effort effectively maintains the LR’s tone (analogous to muscular training). However, when viewing a smartphone, the LR is no longer required to contract as much. Therefore, following long and excessive smartphone use, the muscular training effect of the LR decreases. This loss of LR tone leads to a breakdown of binocular fusion, termed “decompensated esophoria,” which subsequently manifests as esotropia (presence of diplopia) when viewing the distance (bottom right).

The causes of AACE are various, and it is important to rule out severe neurological disorders, including brain tumors, hydrocephalus, or myasthenia gravis [5], though these are rare. Most cases of “smartphone esotropia” are believed to be a different kind of issue and fall under the category of decompensated esophoria, proposed by Von Noorden [6]. Patients with decompensated esophoria perceive diplopia when enhanced divergence fusional amplitudes are no longer sufficient [7]. Therefore, we hypothesized that long and excessive smartphone use can trigger the onset of esotropia (presence of diplopia) in people with this predisposing condition, such as esophoria (absence of diplopia) (Fig 1). If smartphone use caused new esotropia development in people without predisposing conditions, a marked increase in esotropia should be observed. Although proving this hypothesis incontrovertibly is impossible, even a partial verification would require an extremely large cohort study, at the very least.

We previously reported the prevalence and annual incidence of strabismus in Japan in 2019, based on data from the National Database of Health Insurance Claims and Specific Health Checkups of Japan (NDB) [8]. However, we had not yet investigated the temporal change in the annual incidence of esotropia. Therefore, this study aims to examine the changes in the annual incidence of esotropia, as well as the number of strabismus surgeries for esotropia, in Japan between 2014 and 2019—a period marked by a rapid increase in smartphone users.

## Materials and methods

This retrospective, nationwide, population-based cohort study was approved by the ethics committee of the Kyoto University Graduate School of Medicine (Kyoto, Japan; approval ID: R2035). All study protocols adhered to the tenets of the Declaration of Helsinki.

### Database

Similar to previous studies [8–10], we utilized the NDB to count cases of esotropia. Briefly, all claims in Japan have been submitted electronically to the Japanese Ministry of Health, Labour and Welfare (MHLW) since 2011, and the medical data are anonymized and stored in the NDB. We analyzed all data at the NDB Onsite Research Center Kyoto, after receiving approval from the MHLW. To identify the same person across different claims, we used ID 0, a newly generated ID. After linking the claims data of individuals using ID 0, we identified unique ID 0s of individuals who had been diagnosed with esotropia.

### Analysis of annual incidence and strabismus surgery number

We counted the number of cases of newly diagnosed esotropia, defined as having the Japanese health insurance claim codes for AACE-related NDB codes (Table 1), for each year between 2014 and 2019. We did not include congenital esotropia, infantile esotropia, accommodative esotropia, sensory esotropia, and abducens palsy from them, in contrast to a previous report [8], because we considered that they had minimal relevance to AACE.

**Table 1:** Diagnostic codes for esotropia.

NDB diagnostic code	Diagnosis
3780007	Esotropia
8831506	Intermittent esotropia
8842333	A-pattern esotropia
8842334	V-pattern esotropia
8832559	Concomitant esotropia
8833552	Alternate esotropia
8833551	Alternate concomitant esotropia
8832739	Tonic esotropia
8839838	Partially accommodative esotropia
8831023	Divergence insufficiency
8831023	Divergence palsy

We identified the unique IDs of individuals diagnosed with strabismus using the NDB diagnostic codes.

NDB, National Database of Health Insurance Claims and Specific Health Checkups of Japan.

We set three years as a wash-out period (2011–2013), and we did not include data from 2020 onward, as patients refrained from visiting hospitals due to the COVID-19 pandemic. We calculated the incidence (per 100,000 person-years) as the proportion of the number of cases of newly diagnosed esotropia against the corresponding year’s Japanese population, which was obtained from the e-Stat website that shows the Japanese government statistics (https://www.e-stat.go.jp/en). We also calculated each annual change in incidence and increase-decrease rate (%) of esotropia against the last-year incidence (2015–2019). Furthermore, we counted the cases that underwent strabismus surgery, defined as initially having the surgical procedure codes (Japanese original K-codes) for strabismus surgeries (Table 2), for the above-defined esotropia, for each year, and calculated each annual strabismus surgery rate against newly diagnosed cases of esotropia.

**Table 2:** Surgical procedure codes for strabismus surgeries.

Code	Surgery
150083310	Strabismus surgery (advancement)
150083410	Strabismus surgery (recession)
150083510	Strabismus surgery (advancement and recession)
150083610	Strabismus surgery (oblique muscle surgery)
150273910	Strabismus surgery (rectus advancement and recession and oblique surgery)
150083810	Transposition surgery of extraocular muscle

We counted the cases who underwent strabismus surgery, defined as initially having the above codes.

### Statistical analysis

Data are presented as mean ± standard deviation. We calculated the 95% confidence intervals (CIs) for the annual incidence rates based on the Poisson distribution approximation, and performed Spearman’s correlation analyses between the annual incidence of esotropia and the smartphone household penetration, as supplied by the website of the Japanese Ministry of Internal Affairs and Communications (https://www.soumu.go.jp/main_sosiki/joho_tsusin/eng/whitepaper/2021/pdf/chapter-4.pdf) and between the annual incidence and the number of strabismus surgeries between 2014 and 2019 using software of SPSS version 27 (IBM, Armonk, NY, USA). *P* < 0.05 was considered statistically significant.

## Results

**Fig 2:**
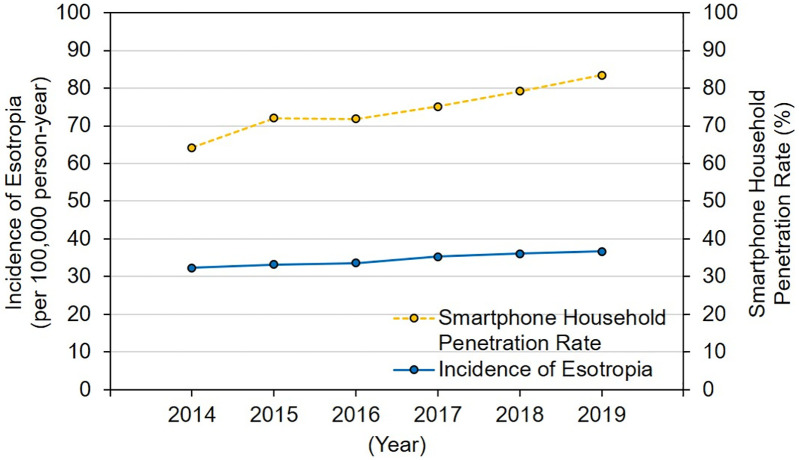
Annual incidence of esotropia and smartphone household penetration rate in Japan. The blue solid line demonstrates the annual incidence of esotropia, and the orange dotted line demonstrates the smartphone household penetration rate, as supplied by the website of the Japanese Ministry of Internal Affairs and Communications (https://www.soumu.go.jp/main_sosiki/joho_tsusin/eng/whitepaper/2021/pdf/chapter-4.pdf). The incidence of esotropia appears to increase annually as the smartphone household penetration rate increases.

The annual cases of newly diagnosed esotropia were 40,997, 42,134, 42,618, 44,816, 45,708, and 46,338 from 2014 to 2019, respectively. Considering the Japanese population, the corresponding annual incidences were 32.26 (95% CI, 31.95–32.57), 33.15 (95% CI, 32.83–33.47), 33.55 (95% CI, 33.23–33.86), 35.31 (95% CI, 34.98–35.64), 36.06 (95% CI, 35.73–36.39), and 36.61 (95% CI, 36.28–36.95) per 100,000 person-years, respectively (Table 3). The incidence increased slightly year by year between 2014 and 2019 (Fig 2), and the mean annual increase of incidence compared to the last-year incidence was 0.87 ± 0.53 (range, 0.39–1.76) per 100,000 person-years. Thus, the mean annual increase rate of incidence against last-year incidence was 2.49 ± 1.62%/year (range, 1.15–5.16%).

**Table 3:** Annual incidence of esotropia and number of strabismus surgeries between 2014 and 2019.

Year	Newly diagnosed cases, n	Annual incidence, per 100,000 person-year (95% CI)	Japanese population, n	Strabismus surgery, n
2014	40,997	32.26 (31.95–32.57)	127,083,000	3,061
2015	42,134	33.15 (32.84–33.47)	127,095,000	3,232
2016	42,618	33.55 (33.23–33.86)	127,042,000	3,191
2017	44,816	35.31 (34.98–35.64)	126,919,000	3,426
2018	45,708	36.06 (35.73–36.39)	126,749,000	3,579
2019	46,338	36.61 (36.28–36.95)	126,555,000	3,743

CI = confidence interval.

Japanese population data are based on the e-Stat website showing Japanese government statistics (https://www.e-stat.go.jp/en).

The annual numbers of strabismus surgeries for cases with esotropia were 3,061, 3,232, 3,191, 3,426, 3,579, and 3,747 from 2014 to 2019, respectively. Thus, the annual strabismus surgery rate against the number of newly diagnosed cases was 7.47–8.08%, which appeared to be fairly constant. Taken together, the overall incidence of esotropia increased, and the total number of strabismus surgeries increased; however, the rate of patients eligible for surgery did not increase.

We found strong and statistically significant correlations between the annual incidence of esotropia and the smartphone household penetration (*P* = 0.005, r = 0.943) as well as between the annual incidence of esotropia and the annual number of strabismus surgeries (*P* = 0.005, r = 0.943).

## Discussion

This nationwide study provides the first population-based evidence that the annual incidence of esotropia is increasing. We found that the incidence in Japan increased from 32.26 to 36.61 per 100,000 person-years between 2014 and 2019. During this period, a major societal change was the rapid popularization of digital devices, and our analysis showed a significant correlation between the annual incidence and smartphone household penetration. We emphasize that this is an ecological study; thus, the correlation at the population level does not establish individual-level causation and may be influenced by other societal changes. However, the 6-year increase rate between 2014 and 2019 was relatively lower in the annual incidence than in smartphone household penetration (13% vs. 30%). Calculating numbers, the smartphone users increased by 36,000,000 people, and the incidence of esotropia by 5,300 patients during the 6 years. Our findings would partly support the hypothesis that excessive smartphone use triggers patients with a predisposing condition, such as esophoria, to develop constant esotropia. This dependency on a predisposing factor likely explains why the increase in esotropia (13%) was lower than the rapid surge in smartphone penetration (30%). Further causal studies are necessary, but would actually be difficult to perform. Furthermore, the mean annual rate of the number of strabismus surgeries against newly diagnosed cases of esotropia remained virtually unchanged across the years; therefore, the disease severity might not change.

The mechanism of the link between esotropia onset and digital device use can be considered from both direct and indirect effect perspectives. First, the direct effect is tied to the way our eyes work to focus on a digital device at a near position (Fig 1). To achieve binocular single vision, our eyes need to turn inward (convergence) and adjust their focus (accommodation). Patients with esophoria naturally have a tendency for their eyes to drift inward. They can maintain straight vision by actively using their outward-turning eyes (divergence). However, when they repeatedly engage in convergence for prolonged periods, as with digital device use, the muscles responsible for divergence can weaken from disuse. This sustained near work exhausts the eyes’ ability to maintain alignment, leading to constant esotropia (known as decompensated esophoria). This mechanism is supported by previous studies showing that this condition can be partially resolved when patients stop using their devices excessively [1,3,4], suggesting that the divergence strength can be restored in the early phase. Crucially, we define “excessive” use physiologically rather than temporally; it denotes prolonged near-work that overwhelms an individual’s fusional divergence amplitude and vergence adaptation capacity, irrespective of a universal hourly threshold. Specifically, ophthalmologists should assess digital device usage history in patients with acute esotropia. We also recommend a trial of digital abstinence to evaluate reversibility before considering surgical intervention, alongside preventive measures such as the 20-20-20 rule [11]. Second, the indirect effects may involve the development of myopia due to excessive digital device use. A meta-analysis study concluded that exposure to smart devices may be associated with an increased risk of myopia [12]. A case-series study reported progressive esotropia with high myopia before strabismus fixus [13]. Therefore, for some patients, excess use of digital devices might first induce high myopia, which then contributes to the development of esotropia.

We considered that another cause of the increased incidence of ET was age-related strabismus, including sagging eye syndrome. However, our data indicate that demographic shifts alone cannot explain the observed uptrend. According to the website of the Japanese Ministry of Internal Affairs and Communications, the elderly population (aged ≥ 65 years) increased by 8.3% from 2014 (33,000,000) to 2019 (35,754,000). In contrast, the annual number of newly diagnosed esotropia cases increased by 13.0% during the same period (from 40,997 to 46,338), outpacing the aging rate by a factor of approximately 1.6. Furthermore, the younger population (aged < 15 years) decreased by 6.0% (from 16,233,000 to 15,259,000) during this period. Despite this demographic “headwind,” the total number of esotropia cases increased substantially. This discrepancy strongly supports the conclusion that environmental factors, rather than population aging, are driving the surge in incidence.

The number of annual strabismus surgeries for esotropia increased in parallel with the rising incidence, with a stable annual ratio of surgeries to this incidence (7.47–8.08%). For comparison, a previous study using the Intelligent Research in Sight Registry in the United States demonstrated that 4.8% of strabismus cases underwent surgery over a four-year period [14]. Although a direct comparison is challenging due to differences in methodology and healthcare systems, our findings suggest that Japan’s surgical rate is relatively high. We considered that this is likely due to Japanese National Health Insurance system, which provides nearly all patients with access to surgery at a low out-of-pocket cost (approximately 70–200 USD). Our findings also contrast with those from England, where a study using the Hospital Episode Statistics of Health and Social Care Information Centre found little change in the total number of strabismus surgeries performed between 2000 and 2014 (1% reduction) [15]. This highlights a potential difference in trends between countries. Further research covering the same period in other nations is needed to better understand these global patterns in esotropia and its treatment.

A major strength of this study is providing a nationwide, population-based account of esotropia incidence in Japan, a dataset of unprecedented scale. Despite this strength, our study has several limitations. First, our findings rely on diagnostic claims data, which were made by various doctors and therefore subject to some diagnostic variability. Although this study did not include “strabismus,” such a diagnosis might include esotropia. We consider the impact of excluding “strabismus” to be minimal because the proportion of “strabismus” among all types of strabismus was small (10.6%; 286,711/2,709,207) in our similar previous study [8]. Therefore, most patients with esotropia would be diagnosed as having esotropia. However, some small degree of underestimation may occur. Regarding detection bias, while a 2016 case series may have raised awareness [1], clinical dissemination takes time. Moreover, the uptrend began in 2014, pre-dating the publication, suggesting detection bias alone cannot explain the increase. Crucially, our use of surgery procedure codes provides a more accurate count, and the uptrend observed in surgical numbers supports the overall trend in incidence. Second, the appropriate wash-out period has not been formally established for this condition. We selected 2014 as the initial year for many similar studies using NDB [16–19]. Furthermore, esotropia is not a disease requiring frequent visits to clinics. Thus, we considered the 3-year wash-out period (2011–2013) appropriate. Third, the stringent criteria for accessing the NDB database limit post-hoc modification of our methodology. Specifically, we could not perform age- or gender-stratified trend analyses because obtaining additional stratified datasets requires a formal re-application and government review process. This procedure is rigid and time-consuming, preventing us from performing additional analyses within the current study framework.

In conclusion, this study provides the first population-based evidence of a significant uptrend in the annual incidence of esotropia and related strabismus surgeries in Japan between 2014 and 2019. The rapid increase in digital device use warrants caution and may be associated with this increase, suggesting a need for clinical guidelines on screen time.
