# Correction: Uptrend in esotropia incidence in the era of excessive smartphone use: A nationwide population-based cohort study in Japan, 2014–2019

**DOI:** 10.1371/journal.pdig.0001532

**Published:** 2026-06-25

**Authors:** Saori Wada, Manabu Miyata, Masahiro Miyake, Ai Kido, Takuro Kamei, Hiroaki Ueshima, Shinya Nakao, Akinari Yamamoto, Kenji Suda, Eri Nakano, Hiroshi Tamura, Akitaka Tsujikawa

In the Discussion section, there is an error in the sixth sentence of the first paragraph. The correct sentence is: Calculating numbers, the smartphone users increased by over 20 million individuals, and the incidence of esotropia by 5,300 patients during the 6 years.
